# 2-[(5-Methyl-1,3,4-thia­diazol-2-yl)sulfan­yl]-*N*′-(4-nitro­benzyl­idene)acetohydrazide monohydrate

**DOI:** 10.1107/S2414314625003645

**Published:** 2025-04-24

**Authors:** Murugan Nidhishree, Sundaramoorthy Gomathi, Jeyaraman Selvaraj Nirmalram, Logesh Mathivathanan

**Affiliations:** a Department of Chemistry, Periyar Maniammai Institute of Science & Technology, Thanjavur-613403, Tamilnadu, India; b Department of Chemistry, Research and Development Cell, PRIST Deemed to be University, Thanjavur-613403, Tamilnadu, India; c Department of Chemistry, School of Advanced Sciences, Vellore Institute of Technology, Vellore-632014, Tamil Nadu, India; University of Aberdeen, United Kingdom

**Keywords:** crystal structure, 1,3,4-thia­diazole, hydrogen bonds

## Abstract

In the title hydrate, C_12_H_11_N_5_O_3_S_2_·H_2_O, the dihedral angle between the aromatic rings is 9.6 (3)°. In the crystal, N—H⋯O and O—H⋯N hydrogen bonds link the components into (101) sheets.

## Abstract 2

In the title hydrate, the dihedral angle between the aromatic rings is 9.6 (3)°. In the crystal, N—H⋯O and O—H⋯N hydrogen bonds link the components into (101) sheets.

## Graphical abstract

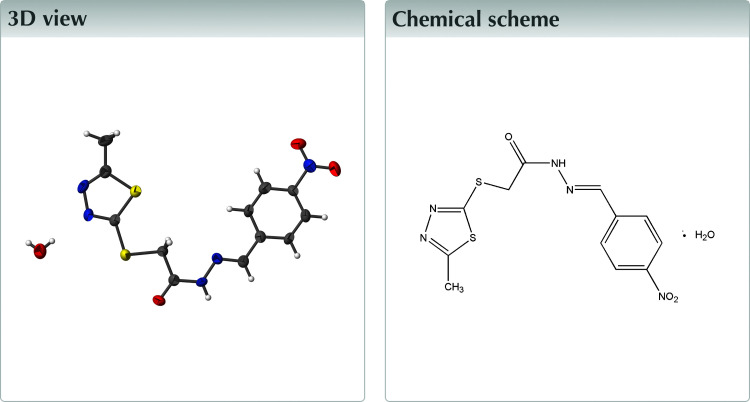

## Structure description

**Figure 1:**
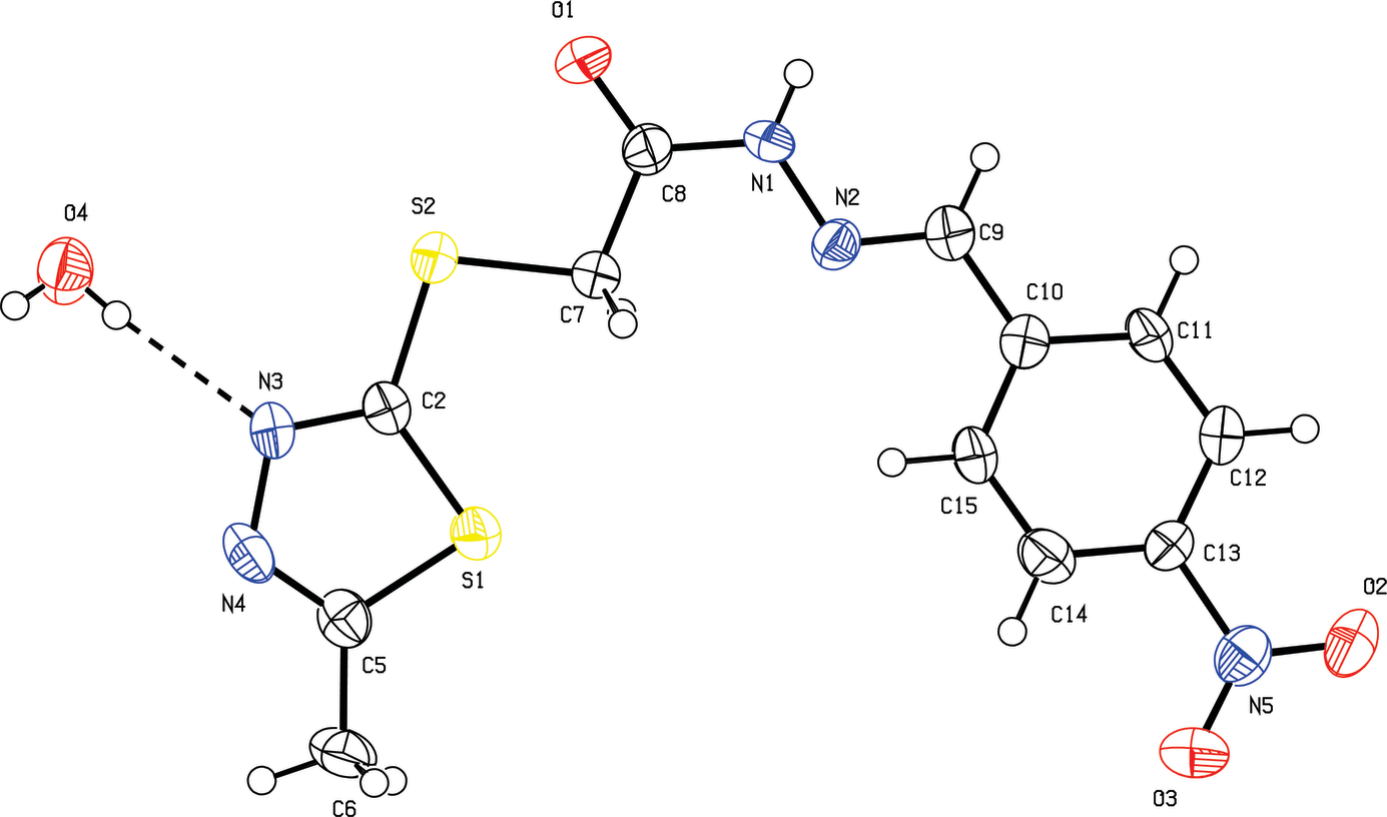
The mol­ecular structure of (**I**) with displacement ellipsoids drawn at the 50% probability level.

1,3,4-Thia­diazole derivatives exhibit various biological activities such as cytotoxic (Janowska *et al.*, 2020 ▸), anti­cancer (Hekal *et al.*, 2023 ▸), anti­convulsant (Luszczki *et al.*, 2015 ▸), anti-epileptic (Anthwal & Nain, 2022 ▸), anti­nociceptive (Altıntop *et al.*, 2016 ▸), anti­tubercular (Jain *et al.*, 2013 ▸), anti­microbial, anti­fungal and anthelmintic activities (Bhinge *et al.*, 2015 ▸). As part of the ongoing studies in this area, the present work describes the synthesis and structure of the title hydrate, C_12_H_11_N_5_O_3_S_2_·H_2_O (**I**) (Fig. 1 ▸ and scheme).

The dihedral angle between the C2/C5/N3/N4/S1 1,3,4-thia­diazole ring and the C10–C15 benzyl­idene ring is 9.6 (3)°. The torsion angles O1—C8—N1—N2, C7—C8—N1—N2 and C8—N1—N2—C9 are 177.1 (5), −2.5 (8) and 174.6 (5)°, respectively. The first and third of these indicate that the mol­ecule adopts a near-planar *trans* conformation. The small value for the second appears to minimize steric hindrance and maintains overall near-planarity. The bond angles for the hydrazide nitro­gen atoms (N1 and N2) are close to 120°, indicative of the expected *sp*^2^ hybridization (Mohan *et al.*, 2011 ▸). Overall, the organic mol­ecule is close to planar (r.m.s. deviation for the non-hydrogen atoms = 0.139 Å).

**Figure 2:**
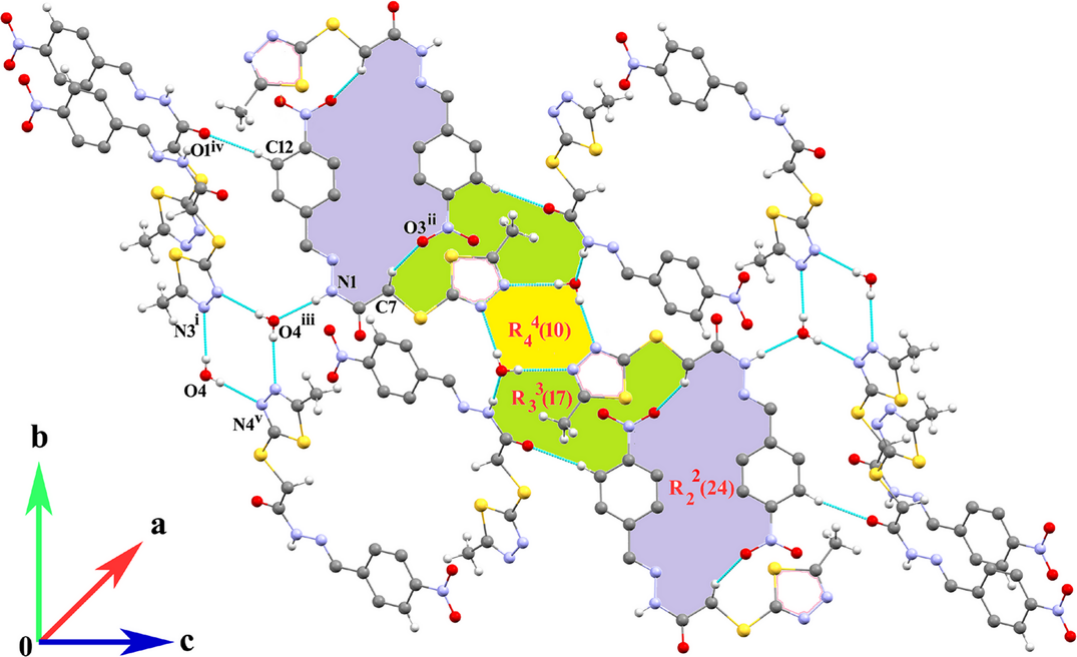
Part of a supra­molecular layer in the crystal of (**I**) showing various supra­molecular motifs. Symmetry codes (i) −1 + *x*, *y*, *z*; (ii) −*x*, −*y*, −*z*; (iii) 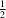 + *x*, 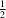 − *y*, −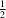 + *z*; (iv) −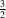 + *x*, 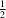 − *y*, −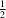 + *z*; (v) 1 − *x*, −*y*, 1 − *z*].

In the crystal, the components are linked by N—H⋯O_w_ and O_w_—H⋯N (w = water) hydrogen bonds (Table 1 ▸), generating infinite (10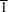) sheets. Various supra­molecular assemblies arise from this connectivity including 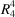
(10) 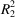
(24), 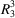
(17), 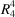
(10), 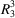
(17) and 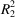
(24) loops (Fig. 2 ▸). Two weak C—H⋯O inter­actions also occur.

The qu­anti­tative contribution of each type inter­action to the Hirshfeld surface is provided in the two-dimensional finger print plots and it is supported by the HS mapped with *d*_norm_. The O⋯H/H⋯O contacts provide a maximum contribution (28.4%) through strong hydrogen bonding, followed by H⋯H (25.2%), and a significant role is played by N⋯H/H⋯N (9.2%) contacts. The other contact types S⋯H/H⋯S (7.7%), C⋯H/H⋯C (8.6%) O⋯C/C⋯O (5.4%) N⋯C/C⋯N (3.9%) and S⋯O/O⋯S (3.4%) presumably play a minor role on the crystal packing of (**I**) (see Figs. S1 and S2 in the supporting information).

## Synthesis and crystallization

The title compound was synthesized by mixing 20 mL of an ethano­lic solution of (5-methyl-[1,3,4]lthia­diazol-2-ylsulfan­yl) acetic acid hydrazide (0.25 mmol) and ethanol:water mixture (3:1 *v*/*v*) 4-nitro­benz­aldehyde (0.25 mmol). The resulting mixture was refluxed under basic conditions for approximately 2 h. The resultant product was dissolved in 20 mL of ethanol, and the solution was allowed to crystallize *via* slow evaporation at room temperature, from which golden crystals of the title compound were harvested.

## Refinement

The crystal data, data collection and structure refinement details for the compound are summarized in Table 2 ▸.

## Supplementary Material

Crystal structure: contains datablock(s) global, I. DOI: 10.1107/S2414314625003645/hb4512sup1.cif

Structure factors: contains datablock(s) I. DOI: 10.1107/S2414314625003645/hb4512Isup2.hkl

The supporting information contains Two dimensional fingerprint plot showing the total contribution of individual types of interactions and HS mapped with dnorm. DOI: 10.1107/S2414314625003645/hb4512sup3.pdf

Supporting information file. DOI: 10.1107/S2414314625003645/hb4512Isup4.cml

CCDC reference: 2445786

Additional supporting information:  crystallographic information; 3D view; checkCIF report

## Figures and Tables

**Table 1:** Hydrogen-bond geometry (Å, °)

*D*—H⋯*A*	*D*—H	H⋯*A*	*D*⋯*A*	*D*—H⋯*A*
N1—H1⋯O4^i^	0.82 (4)	2.08 (4)	2.900 (8)	176 (5)
O4—H4*A*⋯N4^ii^	0.72 (9)	2.32 (9)	3.037 (8)	175 (10)
O4—H4*B*⋯N3	0.75 (8)	2.15 (9)	2.898 (8)	176 (13)
C7—H7*B*⋯O3^iii^	0.97	2.51	3.400 (7)	153
C12—H12⋯O1^iv^	0.93	2.46	3.349 (6)	159

Symmetry codes: (i) 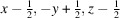; (ii) 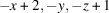; (iii) 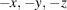; (iv) 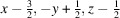.

**Table 2:** Experimental details

Crystal data	
Chemical formula	C_12_H_11_N_5_O_3_S_2_·H_2_O
*M*_r_	355.39
Crystal system, space group	Monoclinic, *P*2_1_/*n*
Temperature (K)	273
*a*, *b*, *c* (Å)	4.9431 (3), 18.3181 (11), 17.2223 (9)
β (°)	95.557 (2)
*V* (Å^3^)	1552.12 (16)
*Z*	4
Radiation type	Mo *K*α
μ (mm^−1^)	0.37
Crystal size (mm)	0.14 × 0.10 × 0.04

Data collection	
Diffractometer	Bruker D8 Quest
No. of measured, independent and observed [*I* > 2σ(*I*)] reflections	7776, 2857, 1703
*R*_int_	0.098
(sin θ/λ)_max_ (Å^−1^)	0.624

Refinement	
*R*[*F*^2^ > 2σ(*F*^2^)], *wR*(*F*^2^), *S*	0.090, 0.155, 1.22
No. of reflections	2857
No. of parameters	221
H-atom treatment	H atoms treated by a mixture of independent and constrained refinement
Δρ_max_, Δρ_min_ (e Å^−3^)	0.35, −0.35

Computer programs: *APEX2* and *SAINT* (Bruker, 2016 ▸), *SHELXT2018* (Sheldrick, 2015*a* ▸), *SHELXL2018* (Sheldrick, 2015*b* ▸), *PLATON* (Spek, 2020 ▸), *Mercury* (Macrae *et al.*, 2020 ▸), *POVRay* (Cason, 2004 ▸) and *publCIF* (Westrip, 2010 ▸).

## full crystallographic data

### 2-[(5-Methyl-1,3,4-thiadiazol-2-yl)sulfanyl]-N'-(4-nitrobenzylidene)acetohydrazide monohydrate Crystal data

**Table:**

C_12_H_11_N_5_O_3_S_2_·H_2_O	*F*(000) = 736
*M**_r_* = 355.39	*D*_x_ = 1.521 Mg m^−^^3^
Monoclinic, *P*2_1_/*n*	Mo *K*α radiation, λ = 0.71073 Å
Hall symbol: -P 2yn	Cell parameters from 2857 reflections
*a* = 4.9431 (3) Å	θ = 2.5–26.3°
*b* = 18.3181 (11) Å	µ = 0.37 mm^−^^1^
*c* = 17.2223 (9) Å	*T* = 273 K
β = 95.557 (2)°	Trapezoid, gold
*V* = 1552.12 (16) Å^3^	0.14 × 0.10 × 0.04 mm
*Z* = 4	

### 2-[(5-Methyl-1,3,4-thiadiazol-2-yl)sulfanyl]-N'-(4-nitrobenzylidene)acetohydrazide monohydrate Data collection

**Table:**

Bruker APEXII CCD diffractometer	*R*_int_ = 0.098
φ and ω scans	θ_max_ = 26.3°, θ_min_ = 2.5°
7776 measured reflections	*h* = −5→5
2857 independent reflections	*k* = −22→19
1703 reflections with *I* > 2σ(*I*)	*l* = −18→18

### 2-[(5-Methyl-1,3,4-thiadiazol-2-yl)sulfanyl]-N'-(4-nitrobenzylidene)acetohydrazide monohydrate Refinement

**Table:**

Refinement on *F*^2^	Primary atom site location: dual
Least-squares matrix: full	Hydrogen site location: mixed
*R*[*F*^2^ > 2σ(*F*^2^)] = 0.090	H atoms treated by a mixture of independent and constrained refinement
*wR*(*F*^2^) = 0.155	W = 1/[Σ^2^(*F*O^2^) + 5.0708*P*] WHERE *P* = (*F*O^2^ + 2*F*C^2^)/3
*S* = 1.22	(Δ/σ)_max_ < 0.001
2857 reflections	Δρ_max_ = 0.35 e Å^−^^3^
221 parameters	Δρ_min_ = −0.35 e Å^−^^3^
0 restraints	

### 2-[(5-Methyl-1,3,4-thiadiazol-2-yl)sulfanyl]-N'-(4-nitrobenzylidene)acetohydrazide monohydrate Special details

**Table:**

Geometry. Bond distances, angles etc. have been calculated using the rounded fractional coordinates. All su's are estimated from the variances of the (full) variance-covariance matrix. The cell esds are taken into account in the estimation of distances, angles and torsion angles
Refinement. Refinement on F^2^ for ALL reflections except those flagged by the user for potential systematic errors. Weighted R-factors wR and all goodnesses of fit S are based on F^2^, conventional R-factors R are based on F, with F set to zero for negative F^2^. The observed criterion of F^2^ > 2sigma(F^2^) is used only for calculating -R-factor-obs etc. and is not relevant to the choice of reflections for refinement. R-factors based on F^2^ are statistically about twice as large as those based on F, and R-factors based on ALL data will be even larger.The nitrogen- and oxygen-bound hydrogen atoms (H1, H14A & H14B) were located from the difference-Fourier map and refined freely. All other H atoms were positioned geometrically and refined *via* a riding model.

### 2-[(5-Methyl-1,3,4-thiadiazol-2-yl)sulfanyl]-N'-(4-nitrobenzylidene)acetohydrazide monohydrate Fractional atomic coordinates and isotropic or equivalent isotropic displacement parameters (Å^2^)

**Table:**

	*x*	*y*	*z*	*U*_iso_*/*U*_eq_	
S1	0.3438 (3)	0.03917 (9)	0.27832 (9)	0.0388 (5)	
S2	0.7558 (3)	0.16604 (9)	0.27385 (9)	0.0406 (6)	
O1	0.8363 (9)	0.2756 (2)	0.1699 (2)	0.0502 (17)	
O2	−0.8321 (10)	0.0733 (3)	−0.2101 (3)	0.0619 (17)	
O3	−0.7278 (10)	−0.0085 (2)	−0.1227 (3)	0.0576 (17)	
N1	0.5049 (11)	0.2563 (3)	0.0730 (3)	0.0344 (17)	
N2	0.2957 (10)	0.2128 (3)	0.0442 (3)	0.0329 (17)	
N3	0.7537 (10)	0.0603 (3)	0.3767 (3)	0.0419 (17)	
N4	0.6281 (11)	−0.0020 (3)	0.4009 (3)	0.0423 (17)	
N5	−0.6936 (11)	0.0505 (3)	−0.1527 (3)	0.0421 (19)	
C2	0.6256 (12)	0.0873 (3)	0.3140 (3)	0.033 (2)	
O4	1.1824 (13)	0.1213 (3)	0.4840 (4)	0.0509 (19)	
C5	0.4153 (12)	−0.0206 (3)	0.3560 (4)	0.038 (2)	
C6	0.2509 (12)	−0.0867 (3)	0.3681 (4)	0.049 (3)	
C7	0.5363 (12)	0.1752 (3)	0.1848 (3)	0.039 (2)	
C8	0.6405 (13)	0.2400 (3)	0.1429 (3)	0.034 (2)	
C9	0.1601 (12)	0.2331 (3)	−0.0185 (3)	0.035 (2)	
C10	−0.0591 (11)	0.1862 (3)	−0.0527 (3)	0.0319 (19)	
C11	−0.2248 (12)	0.2078 (3)	−0.1184 (3)	0.038 (2)	
C12	−0.4307 (12)	0.1647 (3)	−0.1523 (3)	0.039 (2)	
C13	−0.4703 (11)	0.0975 (3)	−0.1198 (3)	0.032 (2)	
C14	−0.3105 (13)	0.0738 (3)	−0.0551 (4)	0.044 (2)	
C15	−0.1059 (13)	0.1177 (3)	−0.0217 (4)	0.042 (2)	
H1	0.557 (9)	0.292 (2)	0.050 (3)	0.006 (13)*	
H6A	0.29906	−0.10554	0.41958	0.0740*	
H6B	0.28606	−0.12305	0.33019	0.0740*	
H6C	0.06142	−0.07423	0.36230	0.0740*	
H7A	0.35003	0.18331	0.19596	0.0460*	
H7B	0.54276	0.13154	0.15321	0.0460*	
H9	0.19963	0.27696	−0.04217	0.0420*	
H11	−0.19521	0.25314	−0.14028	0.0460*	
H12	−0.54010	0.18052	−0.19606	0.0460*	
H14	−0.34078	0.02823	−0.03384	0.0530*	
H15	0.00261	0.10145	0.02197	0.0500*	
H4A	1.231 (18)	0.092 (5)	0.509 (5)	0.10 (4)*	
H4B	1.070 (16)	0.104 (5)	0.458 (5)	0.09 (4)*	

### 2-[(5-Methyl-1,3,4-thiadiazol-2-yl)sulfanyl]-N'-(4-nitrobenzylidene)acetohydrazide monohydrate Atomic displacement parameters (Å^2^)

**Table:**

	*U*^11^	*U*^22^	*U*^33^	*U*^12^	*U*^13^	*U*^23^
S1	0.0353 (9)	0.0392 (9)	0.0406 (10)	−0.0011 (8)	−0.0025 (7)	0.0083 (8)
S2	0.0422 (10)	0.0379 (9)	0.0388 (10)	−0.0074 (8)	−0.0111 (8)	0.0063 (8)
O1	0.056 (3)	0.048 (3)	0.044 (3)	−0.026 (2)	−0.009 (2)	0.004 (2)
O2	0.060 (3)	0.064 (3)	0.056 (3)	−0.010 (3)	−0.023 (3)	0.000 (3)
O3	0.067 (3)	0.041 (3)	0.064 (3)	−0.018 (3)	0.002 (3)	0.007 (3)
N1	0.043 (3)	0.028 (3)	0.032 (3)	−0.010 (3)	0.002 (3)	0.007 (2)
N2	0.033 (3)	0.035 (3)	0.030 (3)	−0.003 (2)	0.000 (2)	−0.003 (2)
N3	0.045 (3)	0.043 (3)	0.035 (3)	−0.003 (3)	−0.010 (3)	0.010 (3)
N4	0.046 (3)	0.045 (3)	0.036 (3)	0.006 (3)	0.005 (3)	0.017 (3)
N5	0.045 (3)	0.044 (4)	0.037 (3)	−0.001 (3)	0.003 (3)	−0.010 (3)
C2	0.035 (4)	0.033 (3)	0.030 (4)	0.003 (3)	0.002 (3)	0.003 (3)
O4	0.061 (4)	0.043 (3)	0.046 (3)	0.006 (3)	−0.009 (3)	−0.004 (3)
C5	0.033 (4)	0.039 (4)	0.041 (4)	0.009 (3)	0.005 (3)	0.005 (3)
C6	0.040 (4)	0.044 (4)	0.065 (5)	−0.005 (3)	0.010 (4)	0.020 (4)
C7	0.043 (4)	0.035 (4)	0.036 (4)	−0.010 (3)	−0.006 (3)	0.007 (3)
C8	0.042 (4)	0.027 (3)	0.031 (4)	0.000 (3)	−0.001 (3)	−0.004 (3)
C9	0.037 (4)	0.037 (4)	0.031 (4)	0.002 (3)	0.002 (3)	0.001 (3)
C10	0.029 (3)	0.037 (4)	0.030 (3)	0.003 (3)	0.004 (3)	−0.002 (3)
C11	0.041 (4)	0.034 (3)	0.038 (4)	0.003 (3)	−0.004 (3)	0.014 (3)
C12	0.039 (4)	0.045 (4)	0.030 (4)	0.000 (3)	−0.006 (3)	0.002 (3)
C13	0.028 (4)	0.034 (4)	0.032 (4)	−0.002 (3)	0.001 (3)	−0.003 (3)
C14	0.048 (4)	0.038 (4)	0.046 (4)	0.000 (3)	0.003 (3)	0.011 (3)
C15	0.041 (4)	0.042 (4)	0.040 (4)	0.001 (3)	−0.010 (3)	0.014 (3)

### 2-[(5-Methyl-1,3,4-thiadiazol-2-yl)sulfanyl]-N'-(4-nitrobenzylidene)acetohydrazide monohydrate Geometric parameters (Å, º)

**Table:**

S1—C2	1.712 (6)	C10—C15	1.392 (8)
S1—C5	1.738 (7)	C10—C11	1.389 (8)
S2—C2	1.749 (6)	C11—C12	1.373 (8)
S2—C7	1.799 (6)	C12—C13	1.374 (8)
O1—C8	1.221 (7)	C13—C14	1.372 (8)
O2—N5	1.220 (7)	C14—C15	1.374 (9)
O3—N5	1.217 (7)	O4—H4A	0.72 (9)
N1—N2	1.361 (8)	O4—H4B	0.75 (8)
N1—C8	1.353 (8)	C6—H6C	0.9600
N2—C9	1.270 (7)	C6—H6A	0.9600
N3—N4	1.383 (8)	C6—H6B	0.9600
N3—C2	1.296 (7)	C7—H7B	0.9700
N4—C5	1.290 (8)	C7—H7A	0.9700
N5—C13	1.470 (8)	C9—H9	0.9300
N1—H1	0.82 (4)	C11—H11	0.9300
C5—C6	1.484 (8)	C12—H12	0.9300
C7—C8	1.506 (8)	C14—H14	0.9300
C9—C10	1.461 (8)	C15—H15	0.9300
			
C2—S1—C5	87.2 (3)	N5—C13—C14	118.7 (5)
C2—S2—C7	101.5 (3)	N5—C13—C12	119.9 (5)
N2—N1—C8	119.3 (5)	C12—C13—C14	121.4 (5)
N1—N2—C9	117.3 (5)	C13—C14—C15	119.8 (5)
N4—N3—C2	111.6 (5)	C10—C15—C14	120.7 (6)
N3—N4—C5	113.7 (5)	H4A—O4—H4B	103 (10)
O2—N5—O3	123.9 (6)	C5—C6—H6B	109.00
O2—N5—C13	117.0 (5)	C5—C6—H6C	109.00
O3—N5—C13	119.1 (5)	C5—C6—H6A	109.00
C8—N1—H1	117 (3)	H6A—C6—H6C	110.00
N2—N1—H1	124 (3)	H6B—C6—H6C	109.00
S1—C2—N3	114.7 (4)	H6A—C6—H6B	109.00
S2—C2—N3	118.4 (4)	S2—C7—H7B	111.00
S1—C2—S2	126.9 (3)	C8—C7—H7A	110.00
N4—C5—C6	123.9 (6)	C8—C7—H7B	110.00
S1—C5—N4	112.9 (4)	H7A—C7—H7B	109.00
S1—C5—C6	123.3 (5)	S2—C7—H7A	111.00
S2—C7—C8	106.0 (4)	C10—C9—H9	121.00
N1—C8—C7	115.9 (5)	N2—C9—H9	121.00
O1—C8—C7	122.3 (5)	C10—C11—H11	119.00
O1—C8—N1	121.8 (5)	C12—C11—H11	119.00
N2—C9—C10	118.7 (5)	C13—C12—H12	121.00
C11—C10—C15	117.6 (5)	C11—C12—H12	121.00
C9—C10—C11	121.1 (5)	C13—C14—H14	120.00
C9—C10—C15	121.3 (5)	C15—C14—H14	120.00
C10—C11—C12	122.5 (5)	C10—C15—H15	120.00
C11—C12—C13	118.1 (5)	C14—C15—H15	120.00
			
C5—S1—C2—S2	178.8 (4)	O2—N5—C13—C14	179.8 (6)
C5—S1—C2—N3	0.1 (5)	O3—N5—C13—C12	−179.2 (5)
C2—S1—C5—N4	0.5 (5)	O3—N5—C13—C14	−1.3 (8)
C2—S1—C5—C6	−178.4 (5)	S2—C7—C8—O1	−0.4 (7)
C7—S2—C2—S1	−4.9 (5)	S2—C7—C8—N1	179.2 (4)
C7—S2—C2—N3	173.8 (5)	N2—C9—C10—C11	175.9 (5)
C2—S2—C7—C8	−177.1 (4)	N2—C9—C10—C15	−5.5 (8)
C8—N1—N2—C9	174.6 (5)	C9—C10—C11—C12	179.4 (5)
N2—N1—C8—O1	177.1 (5)	C15—C10—C11—C12	0.7 (8)
N2—N1—C8—C7	−2.5 (8)	C9—C10—C15—C14	−179.1 (6)
N1—N2—C9—C10	177.6 (5)	C11—C10—C15—C14	−0.5 (9)
C2—N3—N4—C5	1.0 (7)	C10—C11—C12—C13	−0.6 (9)
N4—N3—C2—S1	−0.6 (6)	C11—C12—C13—N5	178.1 (5)
N4—N3—C2—S2	−179.5 (4)	C11—C12—C13—C14	0.3 (8)
N3—N4—C5—S1	−1.0 (7)	N5—C13—C14—C15	−177.9 (6)
N3—N4—C5—C6	177.9 (5)	C12—C13—C14—C15	−0.1 (9)
O2—N5—C13—C12	1.9 (8)	C13—C14—C15—C10	0.2 (10)

### 2-[(5-Methyl-1,3,4-thiadiazol-2-yl)sulfanyl]-N'-(4-nitrobenzylidene)acetohydrazide monohydrate Hydrogen-bond geometry (Å, º)

**Table:**

*D*—H···*A*	*D*—H	H···*A*	*D*···*A*	*D*—H···*A*
N1—H1···O4^i^	0.82 (4)	2.08 (4)	2.900 (8)	176 (5)
O4—H4*A*···N4^ii^	0.72 (9)	2.32 (9)	3.037 (8)	175 (10)
O4—H4*B*···N3	0.75 (8)	2.15 (9)	2.898 (8)	176 (13)
C7—H7*B*···O3^iii^	0.97	2.51	3.400 (7)	153
C12—H12···O1^iv^	0.93	2.46	3.349 (6)	159

Symmetry codes: (i) *x*−1/2, −*y*+1/2, *z*−1/2; (ii) −*x*+2, −*y*, −*z*+1; (iii) −*x*, −*y*, −*z*; (iv) *x*−3/2, −*y*+1/2, *z*−1/2.
