# Supplementary material for: 2-[(5-Methyl-1,3,4-thia­diazol-2-yl)sulfan­yl]-N′-(4-nitro­benzyl­idene)acetohydrazide monohydrate
Source: IUCrdata. 2025 Apr 24;10(Pt 4):x250364. doi: 10.1107/S2414314625003645 (PMC12054755; doi:10.1107/S2414314625003645)
Supplement: Supplementary file 3 [file x-10-x250364-sup3.pdf]

(5- Methyl-[1,3,4]thiadiazol-2-ylsulfanyl)-acetic acid (4-nitro-benzylidene)-hydrazide monohydrate

Murugan Nidhishree[a], Sundaramoorthy Gomathi[a]*, Jeyaraman Selvaraj Nirmalram[b] & Mathivathanan Logesh[c]

[a]Department of Chemistry, Periyar Maniammai Institute of Science and Technology, Thanjavur-613403, Tamilnadu, India.
[b]Department of Chemistry, Research and Development Cell, PRIST Deemed to be University, Thanjavur-613403, Tamilnadu, India.
[c]Department of Chemistry, School of Advanced Sciences, Vellore Institute of Technology, Vellore-632014, Tamil Nadu, India

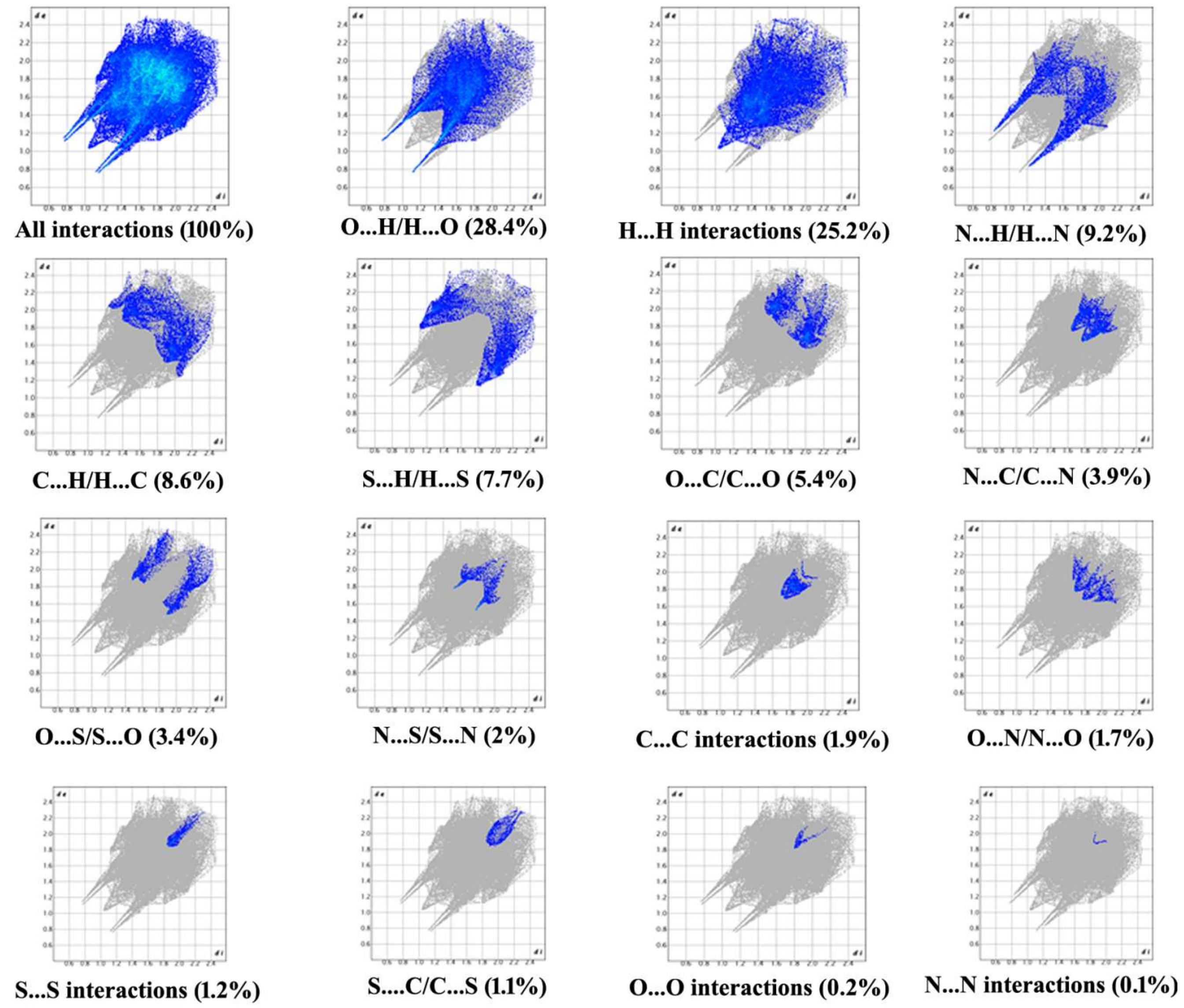

Fig. S1: Two dimensional fingerprint plot of TDNBH showing the total contribution of individual types of interactions

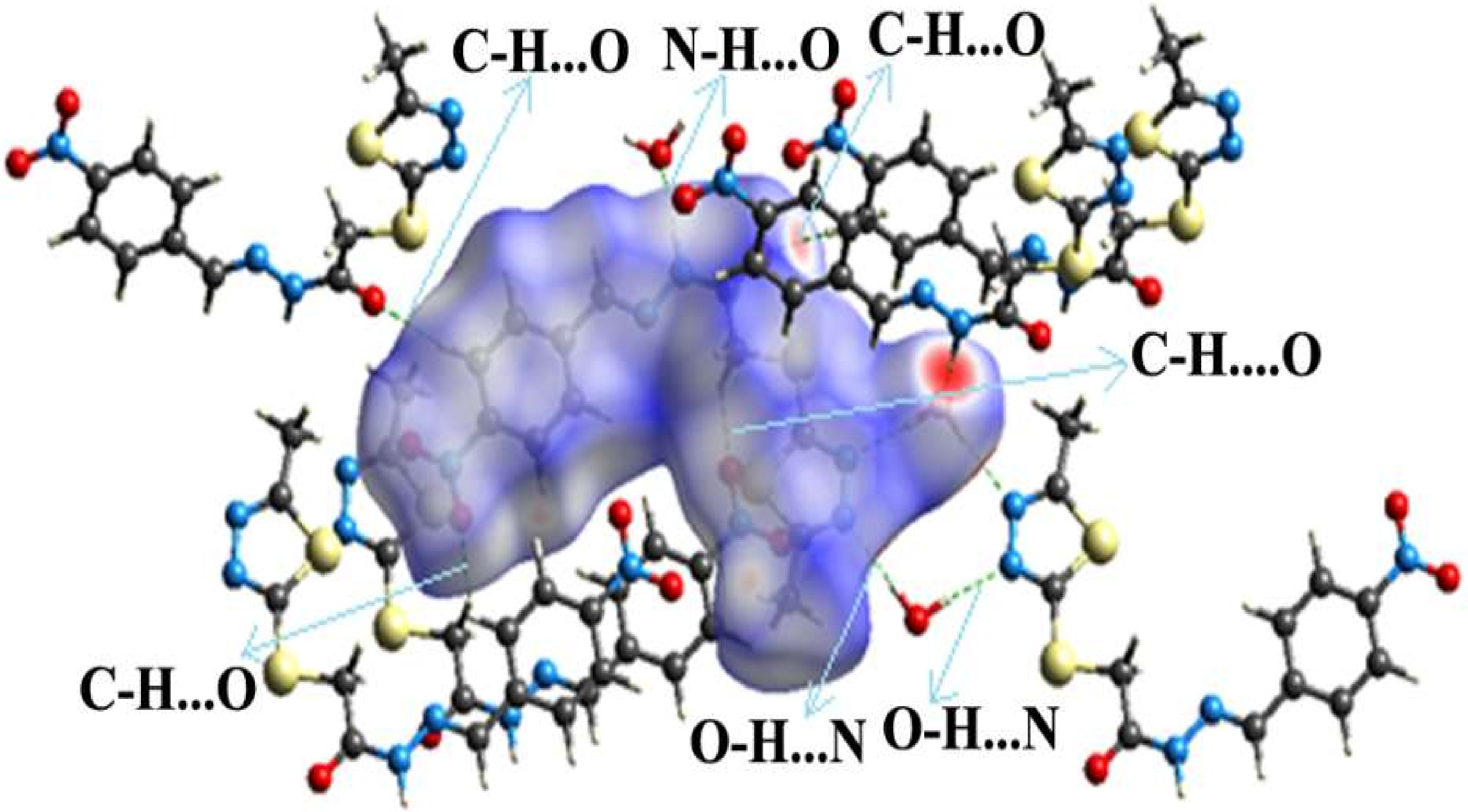

Fig. S2: HS for the compound mapped with d_{norm}
